# MTAP: The Motif Tool Assessment Platform

**DOI:** 10.1186/1471-2105-9-S9-S6

**Published:** 2008-08-12

**Authors:** Daniel Quest, Kathryn Dempsey, Mohammad Shafiullah, Dhundy Bastola, Hesham Ali

**Affiliations:** 1 College of Information Science & Technology, University of Nebraska at Omaha, Omaha NE, USA; 2 Department of Pathology and Microbiology, University of Nebraska Medical Center, Omaha NE. 68182-0694, USA

## Abstract

**Background:**

In recent years, substantial effort has been applied to de novo regulatory motif discovery. At this time, more than 150 software tools exist to detect regulatory binding sites given a set of genomic sequences. As the number of software packages increases, it becomes more important to identify the tools with the best performance characteristics for specific problem domains. Identifying the correct tool is difficult because of the great variability in motif detection software. Consequently, many labs spend considerable effort testing methods to find one that works well in their problem of interest.

**Results:**

In this work, we propose a method (MTAP) that substantially reduces the effort required to assess de novo regulatory motif discovery software. MTAP differs from previous attempts at regulatory motif assessment in that it automates motif discovery tool pipelines (something that traditionally required many manual steps), automatically constructs orthologous upstream sequences, and provides automated benchmarks for many popular tools. As a proof of concept, we have run benchmarks over human, mouse, fly, yeast, *E. coli *and *B. subtilis*.

**Conclusion:**

MTAP presents a new approach to the challenging problem of assessing regulatory motif discovery methods. The most current version of MTAP can be downloaded from

## Background

The regulation of gene expression in the cell is controlled by regulatory proteins and functional RNAs that interact specifically with binding locations on the DNA. In the past century, molecular biologists have developed many innovative techniques to identify sites on the DNA that are bound and regulated by functional RNA and proteins. As the number of sequenced organisms increases, traditional techniques such as gel shift assays in combination with DNAse foot-printing assays can not keep pace with the explosion of available sequences. While accurate, traditional methods require a great deal of time and expertise. Traditional methods also require that the binding energy of the protein or RNA is great enough to maintain contact though the course of the assay. Reductionist approaches can be problematic when faced with non-specific binding sites or with sites that require the formation of a complex with adjacent units in order to function. It is for these reasons that complementary approaches on the computer have been developed in recent years to increase success in discovering and annotating cis-regulatory modules.

Computational discovery of functional subsequences has been around for some time. In 1984, Galas, Eggert, and Waterman proposed a method for finding and characterizing binding positions for the σ 70 protein in *E. coli *promoter sequences [1]. In its most basic form, the problem is identifying sequence signatures, or motifs, that exist in a set of sequences that share the same property (e.g. promoters bound by protein *A*), but does not exist in a set of similar sequences that does not have the same property (e.g. promoters not bound by protein *A*). Computational approaches have the advantage that they can reduce the guess work and cost associated with pure biochemical approaches. Consequently, many computational methods such as Gibbs [2,3], MEME [4], Consensus [5,6], BioProspector [7], AlignAce [8], Ann-Spec [9], MTAP: The Motif Tool Assessment Platform.

Glam [10], and Weeder [11,12] have been developed in recent years. Each of these methods employs different algorithmic insights and scoring functions. The scoring function and algorithm parameters impact the representation and rank of the motifs discovered by the algorithm. In recent years, it has become clear that each of these computational methods has distinct advantages and that the best performance is achieved by trying multiple methods over the same data set. The bioinformatics community is currently seeking to simplify this situation by providing approaches that integrate multiple methods into one tool such as BEST [13] and EMD [14]. Approaches that integrate additional sources of information have also emerged in recent years. For example, approaches such as PhyME [15], PhyloGibbs [16], and WeederH [17] integrate sequences from regulatory regions of related organisms. Other approaches, such as REDUCE [18], integrate expression values from gene expression arrays. In addition, the advent of high density arrays and ChIP-chip technology has necessitated methods that integrate genome wide binding data [19].

While there are many advantages in integrating additional sources of information, the steps required also add additional complexity and cost. Each step along the integration pipeline opens a new question: what is the best way to represent and integrate this new data? One has to wonder if additional data is always better in practice. It could be that for some regulatory modules, the additional data also includes additional noise making it more difficult to recover the binding sites. To make matters more confounding, there currently exist more than 150 methods with thousands of possible pre- and post-processing steps and alternative runtime procedures (we have compiled a list of many popular methods here: ). It is clear that benchmarking technology is needed to map cis-regulatory motif discovery methods to data where the method has the best performance. In other words, given a method *M *and a set of co-regulated genes with regulatory sequences *T *= {*t*_1_, *t*_2_,..., *t*_*n*_}, find a mapping *M *→ *T *with expected performance over some threshold. A *T *that has no method over the threshold is particularly interesting in that such data can tell us more about the current limitations of regulatory motif discovery programs so that we can propose improvements.

In recent years, researchers have begun to solve this problem by creating benchmarking datasets. In 2004, Tompa *et al. *published the first assessment of 13 regulatory motif discovery algorithms over Fly, Human, Mouse and Yeast [20]. This work was seminal in that it provided methods for comparing regulatory motif detection software and that it benchmarked a large number of popular tools [13]. In 2007, Sandve *et al. *improved benchmarking technology over the Tompa dataset using a machine learning approach [21]. While these advances opened the important debate on how to benchmark algorithm performance, it remains to be seen if a selection of so few regulatory binding sites is large enough to form a representative set (The assessment included 8 sites from *Saccharomyces cerevisiae*, 6 sites from *Drosophila melanogaster*, 12 sites from *Mus musculus*, and 26 sites from *Homo sapiens*). In response, Klepper *et al. *proposed a larger test set and used it to evaluate several composite motif discovery tools [22]. This is an important first step in making benchmarking datasets more comprehensive. To date, the most comprehensive test set was over the entire *E. coli *genome [23] using known transcription factors found in RegulonDB [24]. While bacterial transcription regulation is very different than regulation in eukaryotes, the density of the RegulonDB annotation is greater than for any other organism and this coverage provides us with unique benchmarking opportunities. Unfortunately, this assessment only considered 5 tools and neglected to include the tool most successful in the Tompa Benchmark (i.e. Weeder). To date, there are no tools that can assess algorithms that incorporate additional sources of data such as sequences from phylogentically related species.

Currently, cis-regulatory motif tools are assessed using the statistics and methods from gene prediction. However, *in silico *gene annotation differs from cis-regulatory module annotation in that in gene prediction there exists an mRNA library for reference. Unlike regulatory binding assays, mRNA library sequencing is high throughput and thus provides a large and diverse benchmarking dataset for gene prediction tools. Consequently, gene prediction and cis-regulatory module prediction are very different problems. We currently have very few completely annotated regulatory modules and therefore motif prediction tools have very few cases to train. In addition, regulatory modules are thought to evolve at a much faster rate than the genes they regulate [25]. If this theory is true, the diversity of regulatory regions is expected to be far greater than the coding regions they regulate. This means that the parameters, data, transcription module representation and algorithm are all important factors in evaluating motif detection tools.

In this work, we expand upon our parallel architecture for regulatory motif prediction [26] and propose a method for evaluation and discovery of data algorithm mappings. MTAP is a platform that allows one to vary the way scientists process data and algorithm parameters to fine tune the representation of a regulatory module in method *M*. The goal is to discover the best possible mapping of *M *→ *D*. Our platform also integrates phylogentically related regulatory sequences via computing downstream orthologs from other species. It is clear that phylogentic footprinting has great potential, however assessing methods that incorporate sequence from closely related species is not practical or accurate without automation (mainly because of the rapid availability of new organisms: as new organism sequences become available, benchmarks need to change to take into account the new information). For these reasons, there is a place for adaptive and automatic cis-regulatory motif prediction and benchmarking.

## Problem description

**Figure 1:**
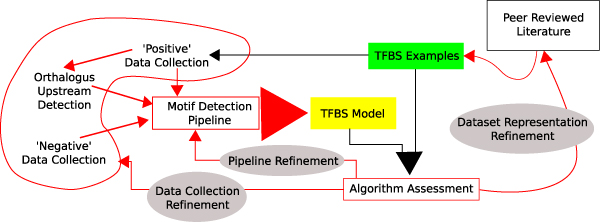
The Motif Tool Assessment Platform (MTAP) enables a researcher to automate each of the steps in cis-regulatory motif discovery, evaluate tools, and propose changes. MTAP is built as a platform where the data collection methods, motif discovery pipelines, and known binding sites are all modular components that can be edited and substituted to look at different aspects of the complex problem of cis-regulatory region annotation.

A cis-regulatory motif discovery pipeline, *M*_*i*_, contains a series of steps to separate transcription factor binding sites from 'background noise'. Motif discovery pipelines require collecting the positive example sequences, collecting negative example sequences, collecting relevant supporting data, running a separation filter to rank relevant sites based on an objective function and finally evaluating or verifying putative sites within the context of each regulatory module. Several factors outside of the pipeline itself contribute to the potential success of the discovery process, mainly: (1) the length of the sequences in the positive and negative sets, (2) the number of sequences in the positive and negative sets, (3) the distribution of transcription factor binding sites in the positive set, (4) the relative entropy of the transcription factor binding motif, and (5) the fraction of null sequences (ones that do not contain a binding site) in the positive set [20]. It is likely there are additional unknown variables that impact the accuracy of an approach. For example, better background sequence models or sequences from closely related species (phylogenetic footprinting) are known to affect performance. Our approach to finding the variables, parameters, and algorithms that are best suited to annotating regulatory regions is an adaptive data collection and analysis platform (Figure 1).

Our hypothesis is that a platform that automates each operation in cis-regulatory motif discovery enables discovery of better methods for cis-regulatory motif prediction. Such a platform enables practitioners and users the necessary flexibility to change characteristics of the data and algorithm parameters, and to isolate and understand known challenge cases. In addition, known methods with many manual steps can be made more explicit. Presently, it is difficult to benchmark existing approaches because they have a great variety of algorithmic parameters that allow the user to interactively optimize the discovery process. While this approach is advantageous because it provides flexibility in the discovery process, it also presents a challenge in algorithm benchmarking: how to formalize data collection, parameter fine tuning, and other intuitive steps taken by the most experienced users of these methods. From a more practical perspective, one has to wonder what evidence experienced practitioners use to determine a set of algorithm refinement steps that will result in better performance over the new dataset (i.e. what qualities in the data indicate a usage procedure). Our hypothesis is that the motif discovery process can be greatly improved if tool parameters are fine tuned based on previous performance benchmarks. We present algorithm benchmarking over a set of known and related transcription factor binding sites (TFBS) as a method to uncover the likely performance on the current dataset. This approach allows the refinement and modification of motif prediction pipelines. In particular, such a benchmarking approach allows for interactive modification of known TFBS in incomplete datasets, modification of the procedures used in collecting positive, negative, and phylogenatically related sequence samples, modification of methods for finding TFBS within the samples, and even modification of the methods used to score motif discovery pipelines.

Our central hypothesis is that by looking at all components of data and software along the cis-regulatory motif discovery process, we can refine our understanding of regulation and discover pipelines with high accuracy. The way we look at this complex problem is a result of how we collect the data, how we build algorithms for discovering regulatory motifs, the biological and manual processes and data structures used to create comprehensive annotations of cis-regulatory regions, and the methods we choose to use in grading these motif discovery tools. We view each of these components as parts that can and should be modified as the specifics of the biological problem at hand become clear. In this paper, we present a open source prototype for cis-regulatory motif discovery algorithm evaluation.

## Implementation

MTAP provides an automated method for ranking regulatory motif detection algorithms. The underlying principle is to create a 'test', *T*, and an 'answer key', *K *= {*k*_1_, *k*_2_,...}. *K *is generated by parsing the raw database management systems (DBMS) of RegulonDB [24], DBTBS [27], PRODORIC [28], RegTransBase [29] and Transfac [30]. The annotated genome, *G*_*j*_, is then parsed for each regulatory binding site annotated in *K*. *T*_*k *_is a collection of sequences corresponding to the surrounding regions of known binding sites for transcription factor *k *in *G*_*j*_. *T*_*k *_is composed of *l *instances of binding positions *T*_1_, *T*_2_,..., *T*_*l *_in *G*_*j *_as annotated by the database. To score a method, *M*_*i*_, we construct an automated pipeline via a scripting language (Perl/Python) that runs the method. Each method has a different algorithmic approach and has different requirements of pre- and post-processing. For each method, we construct background probability models, standardize input datasets, install program dependencies, and provide conversion and utility scripts so that each method can be graded fairly. We consider a fair test to be a test where each program has access to the same information and must mark the transcription factor binding positions in a standard way. This standard marking is then assessed by comparing the annotation of regulatory sites provided by the algorithm with the known annotation in the database.

**Figure 2:**
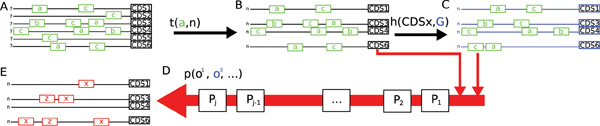
An overview of the MTAP running procedure. A. All known binding positions *G*_*j *_in collected into upstream regions corresponding to each CDS in *G*_*j*_. B. A transformation function *t*(*a*, *n*) creates a test for binding protein *an *bases upstream of each CDS (note that the transcription start site is often unknown or not correctly annotated). C. Background probability information for the entire genome is collected by comparing the upstream regions from the entire genome (or ∀ *k*) to the foreground regions selected by *t*(*a*, *n*). D. Pipeline *p *runs each step of the proposed method *M*_*i*_. E. *M*_*i *_creates a marking on the sequences in B that is evaluated against all marked transcription factor binding positions in B to score the performance of *M*_*i *_in recovering binding sites for transcription factor *a*. This is then repeated for transcription factors *b *and *c*.

A schematic overview of our assessment method is presented in Figure 2. Because databases contain many types of binding positions (not just for transcription factors but also for sigma factor binding sites, ncRNA interaction sites and other binding proteins) these evaluations are an indication of how well each algorithm can recover the binding positions for each element in the regulatory network and not just transcription factor binding positions.

### Generating upstream sequences

**Figure 3:**
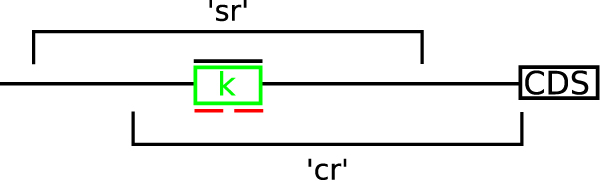
Demonstration of two different methods for obtaining the sequence *n *bases surrounding the known regulatory binding site. In the figure 'cr' refers to completely realistic generation where we find the closest downstream CDS location in the genome and extract *n *bases upstream from that CDS. 'sr' refers to extracting *n*/2 bases upstream and downstream of the center of the binding site. We assume that programs reverse complement sequences appropriately as needed by the method as part of the discovery procedure but provide upstream sequences on the positive strand relative to the downstream CDS. The known motif 'blackfile' sequence is represented by a black line over the binding site *k *that refers to the region that is bound by the transcription factor. The red regions in the diagram illustrate the actual binding positions known for motif *k *for those nucleotides that interact with the regulatory protein.

Varying upstream length allows us to explore the trade off between detecting long range interactions (large *n*) and high prediction accuracy (small *n*). MTAP contains two genome substringing methods for generating upstream sequences as shown in Figure 3. 'Completely-realistic' (cr) data generation is best suited to problems that convert gene lists to binding locations (such as a micro-array). 'Semi-realistic' (sr) data generation is best suited to problems that generate a set of sequences (for example when we know the regions of proposed binding sites). In most cases 'sr' constructed data produces a more fair comparison between pattern finding methods, but is not realistic when the tools are applied to a set of co-regulated loci.

Completely-realistic data generation is most often the natural choice, but is limited because the upstream file may not contain the motif for long range interactions. Also, the 'cr' method may select a different downstream gene as part of the data construction procedure. These two issues are realistic in the cis-regulatory motif discovery process and are representative of current problems in cis-regulatory motif discovery. This method is therefore representative of current methods used in constructing co-regulated upstream sequences. Future work could be done to make automated upstream data generation more sensitive to these types of issues.

**Figure 4:**
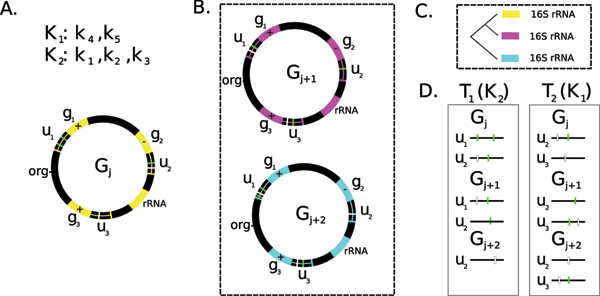
An overview of the pre-processing steps taken in MTAP: A. The source genome *G*_*j *_with genes *g*_1_, *g*_2_, *g*_3 _with known binding sites *k*_1_, *k*_2_, *k*_3_, *k*_4_, *k*_5 _(found clockwise around the genome from the origin); *K*_1 _and *K*_2 _are the known regulatory proteins that bind to the transcription factors B. Two additional genomes, *G*_*j*+1 _and *G*_*j*+2_, that are phylogentically closely related to *G*_*j*_. *G*_*j*+1 _and *G*_*j*+2 _also have binding sites C. The background phylogenetic tree constructed via extracting 16S rRNA D. Two tests *T*_1 _and *T*_2 _corresponding to known positions from *K*_1 _and *K*_2_.

The transformation function *t*(*a*, *n*) applies one of the substring operations (currently 'cr' or 'sr') to the reference genome *G*_*j *_to produce *T*_*k*_. *T*_*k *_is generated by selecting regulator *k *and generating an upstream sequence *u*_*k*, *n *_for all instances of *k *in *G*_*j*_. Figure 4 provides a diagram of several of the stages used to construct *T *= *T*_1_, *T*_2_,... for all instances of *k *in *G*_*j*_. Despite the fact that *T*_*k *_is constructed by considering only TF *k*, all of the other transcription factors in the database are marked and scored if they fall within sequence indices for any sequence in *T*_*k*_.

### Generating orthologous upstreams

After the upstream file, *U*_*k*, *n*_, is constructed, MTAP collects regulatory sequences from closely related genomes *G*_*j*+1_, *G*_*j*+2_,..., by using downstream orthologs. To do this, MTAP constructs a list of all proteins in each genome *G*_*j*+1_, *G*_*j*+2_,.... Before the upstream is created, MTAP uses as orthalog detection method to create an orthalog table, *O *(our current ortholog detection methods are best bi-directional blast hits and RSD [31]). *O *contains a list of protein products from *G*_*j *_and *G*_*j*+1 _and a confidence score *O*_*c *_corresponding to the confidence in the orthology relationship as determined by the ortholog detection method. For each sequence in *U*_*k*_, we select the nearest downstream gene, *g*_*i*, *j *_∈ *G*_*j*_. MTAP then looks up any entries for *g*_*i*, *j *_∈ *O*. If *O*_*c *_> *τ *for some constant *τ*, MTAP appends a region *n *bp upstream of *g*_*j*+1_. If multiple entries exist for *g*_*i*, *j*_, MTAP appends the region upstream *g*_*j*+1, *i *_such that *O*_*c *_is greatest. MTAP continues this procedure for all genomes *G*_*j*_, *G*_*j*+1_,.... This procedure is then repeated to construct *T*_1_, *T*_2_,.... An example result is found in Figure 4D.

Several important points should be made about phylogentic foot-printing. First, the existence of a set of regulatory binding sites exists in *G*_*j *_does not imply the existence of the same set of regulatory binding positions in *G*_*j*+1 _in the same positions. In our example in Figure 4, *T*_*k *_does not contain a binding position for *K*_2 _∈ *G*_*j*+2_. It is possible that our criteria to classify orthologs is too stringent to find an actual ortholog in *G*_*j*+1_. Notice that in our example *T*_*l *_contains no upstream corresponding to *g*_1 _in *G*_*j*+2_. In our example, this occurred because the homology threshold criteria excluded *g*_2 _from *G*_*j*+2_. Again, these problems will exist in any current automated pipeline used for regulatory motif detection. Some of these issues can be resolved within the algorithms themselves: many algorithms incorporate the hypothesis that zero or many instances of the cis-regulatory binding motif exists within the upstream sequence. However, the prospect is very real that we could integrate sequences that are related but do not contain binding sites for transcription factor *k*. Currently, we have very little understanding of the relationship between the evolution of regulatory sequences and coding sequences. It is quite possible that phylogenetically related sequences introduce additional 'noise' with very little signal in regulatory sequences for genes that do not provide critical functions. These complex relationships warrant an in-depth study of regulatory evolution as it relates to coding sequence evolution – which is the subject of future work. For now, we wish to use this approach to benchmark single genome methods as they compare to multiple genome based methods. To our knowledge, MTAP is the first method that allows additional sources of information (sequence conservation) to be automatically integrated as part of the evaluation process.

### Constructing background sequences

Once *T*_*k *_has been constructed for all *K*, we present three possible background sequence files to motif discovery pipelines: (1) all upstream sequences of length *n *in *G*_*j*_, (2) all upstream sequences of length *n *that exist in some *T*_*k*_, and (3) a fasta formated sequence of *G*_*j*_. Programs that incorporate phylogeny have different background sequence requirements. Such programs require a background phylogenetic tree constructed from extracting 16S rRNA from each genome in the study. Some programs require pre-processing steps to calculate an HMM or GC content of the test sequences. Other programs require a background probability distribution of all upstream sequences in *G*_*j*_. We compute each of these requirements for each pipeline in our pre-processing stage and provide the files to each pipeline.

### Compensating for unknown TFBS

Unknown TFBS that exist in *T*_*k *_complicate the assessment process. Tools could predict true sites that are currently unknown or un-annotated. To exclude unknown sites from *T*_*k*_, we construct a Markov chain, *MC*_*m*_, (of depth *m*). For each sequence, *S*_*i*_, in *T*_*k*_, *s*_*i *_is sampled with *MC*_*m*_. We then generate an alternative sequence, *S'*_*i*_, through a random walk through the sates in *MC*_*m*_. For all TFBS in *K *that overlap *s*_*i*_, we insert the true TFBS sequence from *s*_*i *_into *s'*_*i*_. In this way, we use *MC*_*m *_to 'scramble' the upstream sequences in the test and then re-insert the known motifs back into the sequences at the same positions found in the source genome. It is also informative to have instances of true negative sequences produced via *MC*_*m*_, so we produce instances of *T*_*k *_with no inserted TFBS. Orthologous upstream sequences need not be scrambled if they are not scored. These synthetic sequences serve to make a ' more fair' test in those cases where very few of the known motifs are marked. However, such sequences may not correctly incorporate the biological process that generates true sequences. To accommodate this, we also insert TFBS into a random sequence from the set of all upstream sequences in *G*_*j*_.

### Constructing motif discovery pipelines

In constructing motif discovery pipelines, our intention is to include pipelines for as many tools as possible. However, building parsers, installing scripts, and optimizing a pre-processing, post-processing, and runtime pipeline for each of over 150 programs is extremely labor intensive. Our main obstacle is in finding executables that can run on a variety of architectures in a Linux cluster. In many cases, we attempted to contact authors to arrange a port of their tool to a Linux cluster. Many authors are extremely helpful and we would like to thank them for the advice and guidance of how to use and port their tool. However, we realized that even if we have a stand-alone version of a method working on our architecture, MTAP users will still need to install many of the tools directly from the authors (if we do not have the legal authority to distribute the tool). Our strategy for including a tool is as follows:

• Include tools that are the most popular.

• Include tools that present different and novel scoring functions for differentiating background sequences from transcription factor binding sites.

• Include tools that integrate diverse types of information from public sources.

• Do not include tools that do not have a downloadable executable or can not be compiled locally, as such tools can not easily be run thousands of times for assessment purposes.

• Do not include tools that do not have support for different architectures and operating systems (tools we can not run on our computers).

• As we can not redistribute tools with strict licensing agreements or 'abandonware', inclusion of such tools is left to the user community.

Our platform is provided open-source for practitioners who would like to develop their own pipelines to integrate their tool into MTAP. This approach has an advantage in that the developer of the tool is also the developer of the pipeline for evaluating the tool. This could provide an edge as the tool developer will understand the limitations and usage procedures best. We provide our assessment pipelines within our framework for open access review and improvement.

In this work, we developed pipelines for AlignACE [8], Ann-Spec [9], ELPH [32], Gibbs [3], Glam [10], MEME [33], PhyloGibbs [16], PhyME [15], and Weeder [11,12]. For each of these tools in the Tompa *et. al *benchmark, we developed an automated system that was as close to the spirit of the procedure used by the algorithm practitioners as practical. For example, our AlignACE pipeline contains a pre-processing script for calculating GC content of the upstream file and a postprocessing script to mask low complexity repeats using RepeatMasker. The pipeline then parses the raw AlignACE output which results in a ranked list of predicted transcription factor binding sites sorted via the AlignACE MAP score. The pipeline accepts the highest *c *scoring motifs by MAP score and then determines confidence by calculating the group specificity score as provided by CompareACE. A high group specificity score and MAP score indicates a high degree of confidence in the prediction provided by the AlignACE pipeline. For those tools that are not in the Tompa assessment, we carefully followed the usage guides and refined our pipelines using MTAP to produce the best results possible.

For some methods, the code is not available and the method paper does not make it clear if certain data preparation procedures can be accommodated by the method. For example, some methods account for upstream sequences that are reverse complemented while others do not. Some methods account for zero, one, or many motif instances on one strand while others do not. Some methods allow for variable motif widths, while others require an explicit window (thus we need to write a procedure to run the algorithm over all reasonable multiple widths and then rank and combine the results from multiple runs). We assume that motif discovery pipelines are sufficiently robust to account for these technical details and we attempted to make our pipelines robust in this way. However, in constructing these pipelines in this way, we may have overlooked some aspects of the algorithmic approach that would make our pipelines not representative of the original author intent (i.e. our pipeline may not be representative of the best performance possible by expert manual application of the method). We addressed this issue in two ways. First, we built pipelines for multiple implementations of similar approaches (e.g. Gibbs and ELPH). Second, we refined each pipeline based on the objective function found in the literature and the benchmarks obtained via MTAP. To guard against over-fitting of a pipeline to a certain dataset, we did not allow modification of the code or implementation of any procedures not recommended explicitly by the authors. We performed benchmarks over the Tompa assessment datasets, RegulonDB [24], and DBTBS [27] and selected transcription factors at random to validate if the proposed change improved results. As the search space is large, there may exist a set of pre-processing, post-processing, and runtime steps that may improve the performance of our current pipelines. If used in this way, MTAP provides the framework for method developers and method users to formalize and improve motif discovery pipelines.

### Pipeline evaluation

Three levels of specificity can be considered when evaluating the accuracy of *M*_*i*_: (1) *M*_*i *_correctly predicts the region bound by some transcription factor *k*, (2) *M*_*i *_correctly predicts the binding site of *k*, and (3) *M*_*i *_correctly predicts the amino acids in *TF *that interact with specific nucleotides within the regulatory region. *M*_*i *_may also correctly predict the type and strength of the interactions. Predictions of Type 1 are analogous to a gel shift assay in that we can identify a part of the regulatory region bound by a protein. Predictions of Type 2 are analogous to a DNA foot-printing assay. Predictions of Type 2 are more specific in that each region of interaction between *k *and the DNA is identified. For example, if a transcription factor is a dimer, two interaction sites are identified by predictions of Type 2 whereas only one interaction site is identified by a prediction of Type 1 (a site that contains both sites in a Type 2 prediction). The third type of prediction is analogous to determining the crystal structure interaction points of the transcription factor – DNA complex. While the specificity and information provided by the third Type of prediction is far greater than annotations of Type 1 or Type 2, such data is difficult to obtain and few methods make predictions at this level. We therefore generate two annotation files: a 'redfile' and a 'blackfile' corresponding to the site level and region level respectively. To generate an upstream file, we use the blackfile annotation. To assess the performance of an algorithm we evaluate the predictions versus the redfile annotation.

The redfile annotation *RED *= *I*_1_, *I*_2_,... contains a set of intervals *I*_*k *_= (*u*_1_, *v*_1_), (*u*_2_, *v*_2_),... that correspond to the start (*u*_*h*_) and stop (*v*_*h*_) positions in *G*_*j *_corresponding to binding locations for transcription factor *k*. To compare the redfile annotation to the motif tool predictions, *U*_*i *_(upstream sequence *i *in *T*_*k*_) is used as a scaffold to place annotation elements. To annotate the known sites at the nucleotide level, we mark each base, *j*, in *U*_*i *_if *u*_*h *_≤ *j *≤ *v*_*h *_∀ *K*. (*v*_*h *_≤ *j *≤ *u*_*h *_∀ *K *if *U*_*i *_is on the opposite strand) The predicted binding locations, $B_{k,u,v}^{u}$, predicted by *M*_*i *_are parsed and translated into a ranked list of predicted binding sites, each of the form $B_{k1,u1,v1}^{u},B_{k1,u2,v2,...}^{u}$. The ranked list contains elements *TFL*_1_, *TFL*_2_,... sorted according to the confidence that *M*_*i *_has in the prediction accuracy. MTAP accepts the top *c *elements from the rank list for evaluation and inserts them onto the upstream scaffold. MTAP then marks each position, *j*, as a predicted nucleotide if there exists some predicted binding site, *B*, that overlaps it. At the nucleotide level, we collect the overlap statistics shown in Table 1.

**Table 1:** Nucleotide Level Statistics. *u*_*i*, *j *_represents the upstream regulatory sequence *j *at position *i*, *RED *is the set of annotated database positions found in the 'redfile', *B *represents a binding site predicted by method *M*.

Statistic	Definition
nTP	If *u*_*i*, *j *_exists in both *RED *and *B*.
nFN	If *u*_*i*, *j *_exists in *RED *and not in *B*.
nFP	If *u*_*i*, *j *_exists in *B *and not in *RED*.
nTN	If *u*_*i*, *j *_exists in neither *RED *nor *B*.

The first four core statistics (nTP-nucleotide true positives, nFN-nucleotide false negatives, nFP-nucleotide false positives, and nTN-nucleotide true negatives) are collected by summing the number of each of the occurrences shown in Table 1 in *T*_*k*_. The site level statistics (sTP-site true positives, sFN-site false negatives, and sFP-site false positives) are the final three core statistics provided by MTAP. A site level statistic encompasses the idea that a group of adjacent nucleotides marked as binding positions for transcription factor *k *by *M*_*i *_is representative of a binding site annotation. A site is annotated as a true positive if $B_{k,u,v}^{u_{i}}$ overlaps *I*_*x *_∀ *x *by more than τ percent of *I*_*x*_. For example, consider two overlapping sites, a known site *I*_*x *_of 12 consecutive nucleotides and a predicted site *B *of 8 consecutive nucleotides. Given *τ *= .25 If *I*_*x *_shares 3 nucleotides with *B *it is annotated as a sTP. If *I*_*x *_shares only 2 nucleotides with *B *it is annotated as a sFP. Once a site, *I*_*x*_, overlaps a prediction, it can not be annotated as a sFN. All remaining sites, *I*_1_, *I*_2_,..., *I*_*x*_, that do not have an overlapping prediction for tool *M*_*i *_are annotated as sFN. The site level statistics are in Table 2.

**Table 2:** Site Level Statistics.

Statistic	Definition
sTP	Number of known sites overlapped by predicted sites.
sFN	Number of known sites not overlapped by predicted sites.
sFP	Number of predicted sites not overlapped by known sites.

Tompa *et. al *set *τ *to 25%. The logic was that such an overlap makes discovery and refinement of the TFBS possible in the lab. In large scale genome annotations, we find such a threshold to be too strict. For example, many TFBS are not annotated specific enough in databases such as RegulonDB. This results in TFBS that exist in *K *that can be large. Such annotations are actually representative of the region of binding of the transcription factor (a blackfile annotation) and not the binding sites (a redfile annotation). Because many motif discovery programs have fixed motif widths (e.g. 8), a threshold of 25\% would not be sufficient to mark a sTP (e.g. a site of width 60 and a site prediction of length 8). We could choose to rank site level motifs based on a percentage of the prediction width instead of the regulatory motif width, but this would give an unfair advantage to methods that predict larger sites. Our current approach is to set τ equal to the maximum annotated site width in the dataset divided by the minimum expected motif width predicted by our suite of programs (usually 8) times 25%. Our logic is that a degree of overlap indicates that computational and biological refinement of site predictions can still find the site. That said, manual curation of datasets to ensure binding site annotations rather than region annotations is necessary. Standards across regulatory binding site databases to delineate each of the three levels of biological data would greatly increase evaluation accuracy of motif discovery tools. Also, as not all tools provide a mapping for a set of sites to a putative regulator, these statistics are currently not reflective of which regulator is annotated by a site level prediction.

**Figure 5:**
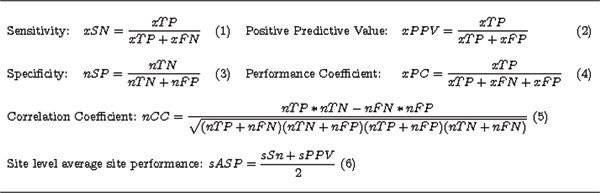
Statistics for Evaluating Motif Prediction Algorithm Implementations.

Following Tompa *et. al *we define the statistics in Figure 5 to perform the assessment.

These statistics enable us to determine the quality of algorithm predictions and therefore infer which tools may be best suited to discover unknown motifs under similar situations. MTAP evaluates each of these statistics by comparing the predictions found in the program output with a set of known binding positions of the same type. For each instance found in the known dataset, a motif prediction tool is run and then parsed. The prediction is compared to the known binding site via the seven key statistics in Table 1 and Table 2. These statistics will then be used to assess the overall performance of the algorithm. MTAP produces an output file for each regulatory binding motif in *K*. Users can sum the raw statistics in these files as they see fit. For this paper, we collect the seven raw performance statistics for each motif in the assessment and then sum these values as if the collection of runs was actually one run.

In some cases, such sums do not graphically represent the contribution of each element in the set to the total performance score. To address this, we also developed a graph that iterates over all runs in the test (*T*_1_, *T*_2_,..., *T*_*k*_) The graph produced is a modified receiver operating characteristic (ROC) curve that combines statistics from multiple runs [34]. We use the following algorithm to produce our ROC graphs:

   for each motif in the dataset:

      input xTP, xFP, nFN, nTN, sTP, sFP, sFN

      P < -calculate xSP and xSN for this motif

   for all motifs in the dataset:

      totalSP = sum(xSP)

      totalSN = sum(xSN)

   sort(P.xSN)

   for all ties in P.xSN; sort(P.xSP)

   for i in P:

      plot(xSN/totalSN, xSP/totalSP)

This produces a curve that travels straight up and then to the right if all motifs in the dataset are predicted correctly. The curve will travel straight to the right and then up if very few of the motifs are predicted correctly. Finally, if the tool predicts sites correctly as often as it predicts sites incorrectly, a line along the diagonal of the ROC graph will be plotted. However, unlike machine learning algorithms where such a graph is often no better than a random classification of sites, using this method there is some value in graphs along the diagonal because we only allow 3 site predictions to be placed on the scaffold.

### Known motif databases

In MTAP, we have implemented interfaces to each of the following databases: RegulonDB [24], DBTBS [27], PRODORIC [28], RegTransBase [29] and TRANSFAC [30] (via the Tompa Benchmark Set). Each of these databases was constructed with different goals and none were built explicitly to evaluate motif prediction tools. Therefore, some database cleaning is required to make these datasets more appropriate for algorithm assessment. We provide two procedures: (1) We require that the known binding site occur in at least *nl *locations in *G*_*j *_(we set this to 3 for the results below). MTAP will not create upstream files for TFBS that do not meet this threshold. (2) We provide a script to analyze multiple sequence alignments and information content of a set of known motifs. We do not require that the TFBS have a consensus sequence as annotated by the database – our logic being that users can eliminate sites without a strong consensus from their analysis by using our information content script and keeping only those sites that exceed a set threshold.

Duplicate instances of the same upstream file are eliminated to prevent bias before they are processed by the programs (however the originals are stored for those users wishing to do further analysis). MTAP does not accept TFBS that do not contain a start and end position in *G*_*j*_. For TFBS that are inconsistent, users can eliminate the sites and re-score the TFBS. Our primary goal in collecting data is to provide automated methods that can improve as the datasets become more comprehensive. At this time, both PRODORIC [28] and RegTransBase [29] have very few TFBS with enough binding positions in the same genome *G*_*j *_for us to provide a comprehensive benchmark (the goal of these databases is more focused on tracking conversation of TFBS across species). Though labor intensive, high annotation density in datasets such as these provides the greatest insight into evaluating computational methods that predict transcription factor binding sites.

## Results

In this section we will provide illustrative examples of how our benchmarking technology can be used to evaluate several important parameters in cis-regulatory motif discovery. To construct a series of tests for evaluation, we extracted 2247 known transcription factor binding positions from RegulonDB [24] corresponding to known positions from *Escherichia coli K12*, and 680 known motifs from DBTBS [27] corresponding to positions from *Bacillus subtilis*. Then, we extracted 522 positions from Eukaryote dataset developed by Tompa *et al.*[20]. Because RegulonDB has the most comprehensive coverage, we used RegulonDB to assess the impact of including two additional genomes (*E. coli *strain W3110, and *E. coli strain *UTI89 in addition to *E. coli *K12). We used our approach to include regulatory regions from these strains in evaluating PhyME and PhyloGibbs. Our primary goal is to show how MTAP can be used to evaluate each of the central questions in cis-regulatory motif discovery. To illustrate the capabilities of MTAP we will use it to evaluate the impact of four of the key factors for regulatory motif discovery introduced earlier in this paper, mainly: (1) the length of the sequences in the positive and negative sets, (2) the number of sequences in the positive and negative sets, (3) the distribution of transcription factor binding sites in the positive set, (4) the relative entropy of the transcription factor binding motif. Through these illustrative examples we intend to show the exploratory power of MTAP towards discovering *M *→ *T *mappings.

### Benchmark automation

**Figure 6:**
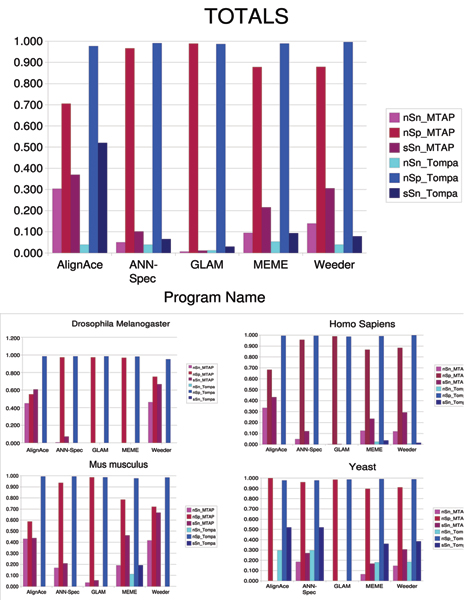
8 performance statistics for 9 motif prediction pipelines generated by MTAP over RegulonDB 400 bp upstream regulatory sequences.

Our first goal was to illustrate that our system of automation can provide similar results to manual runs. To do this, we downloaded the benchmark results from the Tompa assessment and compared these results to results obtained from our pipelines for AlignACE, Ann-Spec, Glam, MEME, and Weeder. Results for nSn, nSp, and sSn for our platform versus the Tompa benchmarks are shown in Figure 6. Overall, sensitivity over our dataset is higher, and specificity suffers slightly. There are a few reasons for this. First, occasionally experts in the Tompa assessment pick a TFBS that is not the highest scoring motif. We think they do this because of their experience with known TFBS in Transfac. Also, MTAP allows the top *c *(three in this case) predictions to be scored as suggested as an improvement by Tompa to increase sensitivity. The original assessment only allowed the top prediction to be scored. In many cases, high specificity is obtained by the tool not making a prediction.

Consequently, we feel pipelines that increase sensitivity at a low cost to specificity provide a good trade-off. Overall performance is similar over this dataset. This provides evidence that our automation pipelines work well relative to manual runs by experts. However, there are still many things better understood by the experts and we continue to refine our pipelines as more information becomes available.

### Automated assessment

**Figure 7:**
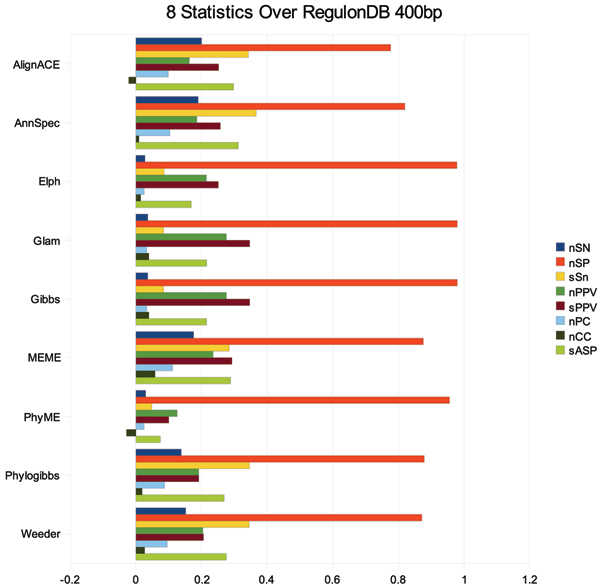
8 performance statistics for 9 motif prediction pipelines generated by MTAP over RegulonDB 400 bp upstream regulatory sequences.

We next used MTAP to produce a benchmark over the sites annotated in RegulonDB. We chose to run MTAP in 'cr' mode over upstream sequences of 400 bp (shown in Figure 7). Overall specificity over this dataset is quite high. This data indicates that tools such as MEME and Weeder achieve higher sensitivity (at both the site level and nucleotide level) without substantial losses to specificity on *E. coli *TFBS. Summed over all TFBS, nCC ranged from -0.03 for PhyloGibbs to 0.06 for MEME. ELPH and Ann-Spec showed the least correlation in this test with a nCC value of 0.01 each. Overall correlation is extremely weak for any tool in the test.

Positive predictive value ranged from 0.13 (PhyME) to 0.28 (Glam) at the nucleotide level and 0.1 (PhyME) to 0.35 (Glam) at the site level. The low values for nPPV and sPPV for PhyME can be attributed to the large number of sites that PhyME did not predict any binding positions. This behaviour is most likely explained by the large amount of sequence conservation found between the upstream regions in these different strains of *E. coli*. It is likely that the multiple sequence alignment step employed by PhyME did not encounter enough sequence divergence in this test set to distinguish between regulatory binding positions and background sequence conservation. The PhyloGibbs algorithm did not appear to encounter the same difficulties. However, PhyloGibbs did not appear to gain a substantial performance gain over Weeder even though it had regulatory sequences from related strains and Weeder did not. AlignACE and Ann-Spec differ from the other programs in that they sacrifice specificity slightly for increased sensitivity. Over many of the regulatory regions both AlignACE and Ann-Spec provided correct predictions somewhere in the list of predicted sites when other tools did not.

Figure 7 provides additional evidence that implementation and user parameters are not as important as the algorithm approach and discrimination function. In this graph, the two programs (ELPH and Gibbs) that use weight matrices for motif discrimination and gibbs sampling as the algorithmic optimization procedure have almost identical performance profiles despite the fact that the parameters provided to each of these tools are quite different in our pipelines.

In the original Tompa Assessment, Weeder had more discrimination power than other approaches. While still quite good, Weeder does not appear to have the same advantages in this test. We feel that this gives further evidence that the organism and type of regulatory mechanism greatly impact the expected performance of a tool.

### Number of upstream sequences

**Figure 8:**
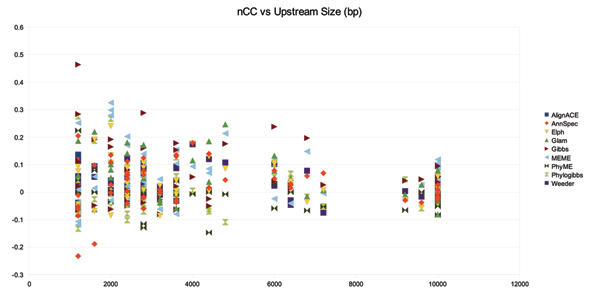
nCC versus total number of base pairs in *T*_*k *_over RegulonDB 400 bp sequences.

As the upstream size increases relative to the size of the transcription factor binding sites, the background signals found in the dataset also increase. This makes discrimination of true transcription factor binding positions more default as the number of regulatory regions and length of each region increases. We wanted to explore the relationship over known transcription factor binding sites. To discover the relationship between |*T*_*l*, *i*_| and |Σ_*i *_*T*_*l*, *i*_| we set |*T*_*l*, *i*_| = 400 *bp *and ran pipelines for AlignACE, AnnSpec, Elph, Glam, Gibbs, MEME, PhyME, Phylogibbs, and Weeder (the notation |*x*| means the sequence length of *x*). Figure 8 shows nCC versus |Σ_*i *_*T*_*l*, *i*_|. As the number of sequences increases, we expect the absolute value of nCC for a tool to increase once the number of co-regulated sequences containing the same signal surpasses some threshold. Once the signal is detected and we continue to increase the number of sequences in the upstream dataset, the absolute value of nCC should decrease as the 'noise' introduced for each added sequence far exceeds the signals. If this is the case for the regulatory regions in *E. coli *K12, then the data indicates that 3 co-regulated sequences provides enough signal to be detected by many of the tools tested in this assessment. While nCC is quite low; the performance over this test set does not indicate that regulatory binding sites are more easily detected if we have more instances of them (as would be suggested by statistical learning theory; e.g. if we have more recorded instances of a phrase uttered by more people an HMM can detect the phrase more easily). It could be that global regulators that have more binding positions over the genome also have more variability in their binding sites. This makes sense if one considers that each instance of a global regulatory binding site must have a different binding energy to control each of the many genes regulated at different rates. The regulatory binding positions with the most occurrences indicate a higher nCC averaged over the motif detection tools. This could be explained by a superior ability of the algorithms to recognize transcription factor binding sites once the number of binding instances is large relative to the length of *G*_*j*_.

**Figure 9:**
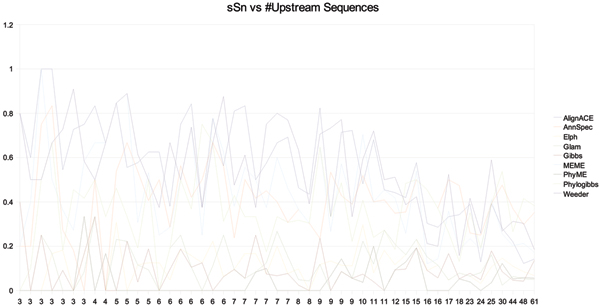
Site sensitivity versus the number of sequences in the upstream file for each *T*_*k *_in RegulonDB with more than 2 unique binding positions in *G*_*j*_.

**Figure 10:**
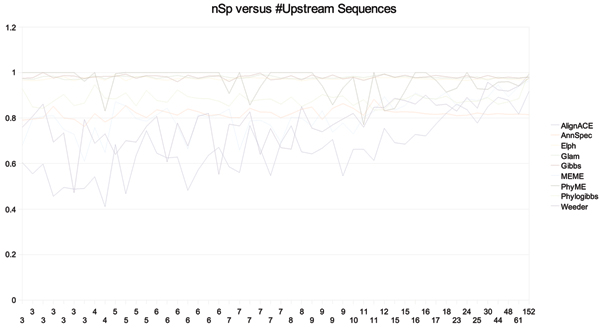
Nucleotide specificity versus the number of sequences in the upstream file for each *T*_*k *_in RegulonDB with more than 2 unique binding positions in *G*_*j*_.

Figures 9 and 10 show the site level sensitivity and nucleotide specificity for AlignACE, AnnSpec, Elph, Glam, Gibbs, MEME, PhyME, Phylogibbs, and Weeder with |*t*_*k*, *i*_| = 400 *bp*. Overall, specificity of these tools maintains a consistent level or increases as the number of sequences in *T*_*k *_increase. The inverse is true for sensitivity. As the number of sequences increase, increased instances of regulatory signal does not lead to increased tool sensitivity. This data indicates that as the number of regulatory signals in the foreground increases linearly, the background 'noise' increases quadratically. High specificity is most likely the result of increased reluctance on the part of tools to make predictions as the number of sequences increases. As the number of sequences increases, the number of co-occuring motif instances also increases. This makes it more likely that multiple occurrences of motif for a related transcription factor may occur in the same upstream set. This motif cross-talk may play a significant roll in defeating current detection methods.

### Length of upstream sequences

To further explore the impact of the size of *T*_*k*_, we used MTAP to extract 500 bp and 200 bp upstream regions from motifs found in RegulonDB and ran pipelines for each of the tools. Most all of the tools did not show any significant correlation between size of *T*_*k *_and prediction performance (data not shown). We believe that this indicates a problem with these classification algorithms over the datasets and not to variation in the size of *T*_*k*_. It could be that these algorithms only classify certain subclasses of regulatory binding profiles accurately. Here we present the results from Weeder to demonstrate the impact of varying length of the upstream file. For Weeder, there is a slight impact on performance if the size of *T*_*k *_is varied. To demonstrate this point, we calculated sensitivity and specificity over the 10 largest and smallest upstream files at 200 bp and 500 bp, respectively (Tables 3 and 4). This data shows that Weeder nucleotide specificity is not greatly impaired by the size of the dataset in these tests. However, we do see a marked decrease in sensitivity both at the nucleotide and site level given larger datasets. The largest dataset has an average sSp of 0.41 while the smallest dataset has a average sSp 0.58 – a substantial difference. While nSn increases as a trend from smaller to larger tests, the predicted window size is on average much smaller than the motif size resulting in many missed predicted nucleotides. Weeder predicts individual transcription factor binding locations can be detected fairly well up to 33066 bp over this dataset. Increasing the dataset size further precipitates a steady drop in sensitivity until predictions are no longer useful.

**Table 3:** Sensitivity and specificity for the 10 largest and smallest datasets from a Weeder run over all of RegulonDB at 500 bp.

10 Smallest Motif Datasets			
Size	nSn	nSp	sSp
1503	0.35	0.81	0.5
1503	0.12	0.92	0.33
1503	0.26	0.82	0.67
1503	0.24	0.79	0.5
1503	0.34	0.81	0.67
1503	0.23	0.61	0.67
2004	0.32	0.62	0.73
2004	0.14	0.72	0.5
2004	0.31	0.78	0.5
2004	0.19	0.74	0.5

Avg:	0.25	0.76	0.56
10 Largest Motif Datasets			
Size	nSn	nSp	sSp

7515	0.1	0.79	0.31
8016	0.4	0.84	0.75
10521	0.34	0.73	0.54
14028	0.25	0.91	0.44
16533	0.18	0.89	0.34
17535	0.15	0.87	0.48
19038	0.11	0.92	0.24
22044	0.1	0.87	0.29
33066	0.13	0.83	0.43
63126	0.09	0.93	0.28

Avg:	0.18	0.86	0.41

**Table 4:** Sensitivity and specificity for the 10 largest and smallest datasets from a Weeder run over all of RegulonDB at 200 bp.

10 Smallest Motif Datasets			
Size	nSn	nSp	sSp
603	0.27	0.65	0.5
603	0.4	0.76	1
603	0.06	0.91	0.33
603	0.06	0.79	0.14
603	0.19	0.85	0.5
603	0.18	0.81	0.33
804	0.47	0.66	0.88
804	0.27	0.84	0.57
804	0.08	0.88	1
804	0.25	0.86	0.5

Avg:	0.22	0.80	0.58
10 Largest Motif Datasets			
Size	nSn	nSp	sSp

3015	0.3	0.67	0.65
3216	0.47	0.84	0.81
4221	0.43	0.87	0.64
5628	0.31	0.82	0.61
6633	0.24	0.81	0.46
7035	0.28	0.78	0.82
7638	0.25	0.77	0.54
8844	0.42	0.74	0.72
13266	0.15	0.89	0.37
25326	0.16	0.91	0.42

Avg:	0.30	0.81	0.60

**Figure 11:**
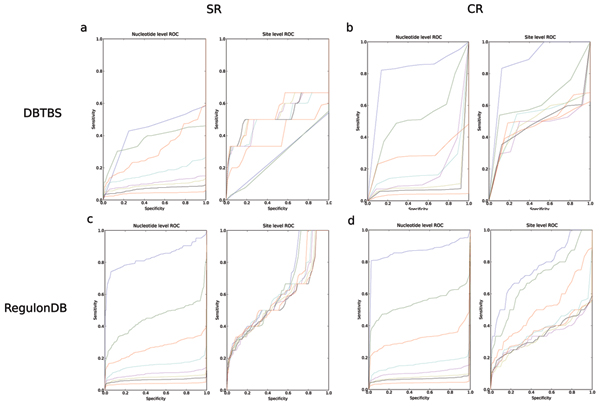
ROC curves for Ann-Spec (20 (blue), 50 (green), 100 (red), 200 (cyan), 300 (magenta), 400 (yellow), 500 (black), and 800 (lower red) basepairs upstream of the CDS.

To further understand the impact of upstream length on motif detection performance. For each tool we ran MTAP and generated ROC graphs for lengths 20 bp, 50 bp, 100 bp, 200 bp, 300 bp, 400 bp, 500 bp, and 800 bp upstream of the gene for DBTBS and RegulonDB. To understand the roll of data generation methods, we generated both completely-realistic ('cr') and semi-realistic ('sr') data. Here we provide the results for ANN-Spec in Figure 11 which is illustrative of these results. The most important characteristic of note is between the performance curves of DBTBS (Figure 11a and 11b) and RegulonDB (Figure 11c and 11d). Figure 11c and 11d are more smooth than Figures 11a and 11b. This is because the number of sites in the regulonDB dataset is much greater than the number of sites annotated in DBTBS.

Commonly, researchers would like to know what motif discovery program is best suited to a particular organism. These findings suggest that this question can not be addressed currently because of the different amounts of coverage found in each dataset. If the coverage of TFBS over the genome were greater in DBTBS, the curves in Figure 11 would show an accurate comparison of the sensitivity-specificity tradeoff in running the Ann-Spec pipeline on each organism. Figure 11a and 11c refer to semi-realistic data generation (the known binding site is in the middle of the upstream sequence). As the window size increases, there is a precipitous drop in Ann-Spec's ability to correctly recover the site. High nSn and nSp are expected at 20 bp 'sr' as most any prediction will overlap the true TFBS. As the window size increases, we expect the performance to remain the same for tools with high recovery rate, but performance should decrease for tools that have poor accuracy. Figure 11c shows a performance drop in nSn and nSp as the length of the upstream sequence increases.

Ann-Spec does appear to recover many sites regardless of the upstream length as noted by the close cluster of performance graphs on the right hand side of Figure 11c. Although similar, 'cr' generated data appears to have higher recovery rates for short windows. We believe that these recovery rates are most likely related to the relationship between the location of the signal for the TFBS and the location of the signal for the *σ*70 binding site – but this requires more exploration.

### Site distribution

**Figure 12:**
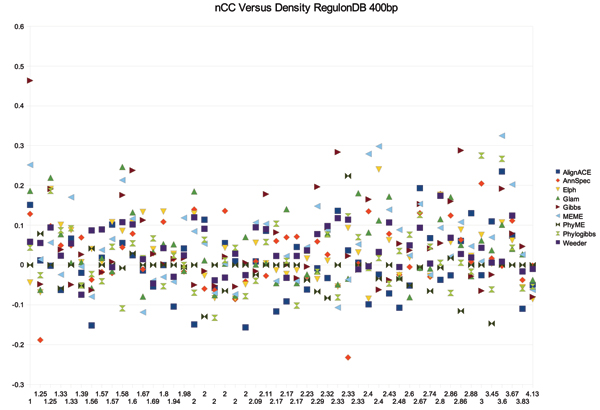
Density of the binding sites in *T*_*k *_versus nCC.

Algorithm practitioners commonly work from the assumption that TFBS have more information than the surrounding sequence. If this is so, the total number of TFBS in *T*_*l *_(or the site density) should impact the performance of a tool. One would expect that a low density of sites would result in higher recovery rates (and vice versa for a high density of sites). To test this, we computed the total number of sites in *T*_*l *_∀ *l *as annotated by *RED*. The site density for *T*_*l *_is the number of sites from *RED *that exist in *T*_*l *_over the number of sequences in *T*_*l*_. We graphed sSn, nSp and nCC versus density for each of the tools. For all of the tools, site density did not appear to have any effect on sSn, nSp and nCC over 400 bp upstream sequences from RegulonDB. Figure 12 shows no apparent decrease in nCC as the site density increases. It could be that there does not exist enough complex regulatory regions in *E. coli *to notice an impact on performance. It is likely that we would see a different result for organisms with more complex regulatory mechanisms, so we can not rule out site density as a factor in accurate regulatory motif prediction. These results do indicate that the 'background' signal is far more complex than originally thought and every program has difficulty distinguishing the foreground TFBS from interfering background signals.

### Site entropy

**Figure 13:**
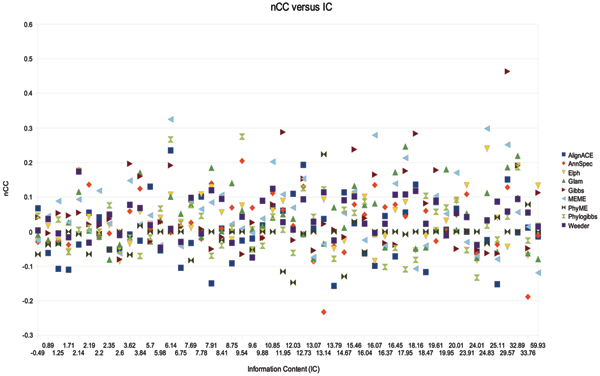
nCC versus Information Content (IC) for *T*_*l *_400 bp from RegulonDB.

**Figure 14:**
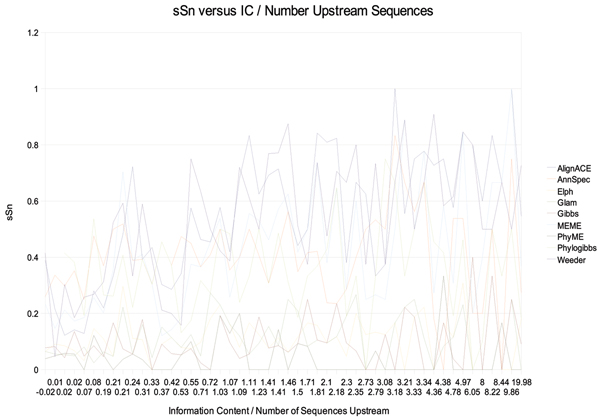
sSn versus information content divided by number of upstream sequences.

If the background signals are simple and the binding sites are complex because they must be conserved by evolution, the relative information content of the TFBS should be greater than the information content found in the background signal. If this is the case, there should be a relationship between the information content of the binding site and prediction accuracy. To test this, we calculated information content for each site in RegulonDB using BioPython and plotted it against nSn, nSp, sSn, (not shown) and nCC (Figure 13). Information content of the site alone does not appear to be a determinative factor in how well these programs can recover the site. Perplexed by this result, we plotted nSn, nSp, sSn, and nCC versus information content divided by the number of upstream sequences in *T*_*k *_(total number of bp in *T*_*k*_). The result for sSn is shown in Figure 14.

Figure 14 shows that for some tools, the ratio of information content of the TFBS to the number of sequences in the upstream file can play a roll in sSn. Stronger information content and less background information implies better performance for Weeder, MEME and AlignACE. For the other tools, it is not clear if this relationship is present.

## Discussion

The most practical outcome of this work is an ability to rank motif prediction tools based on a known TFBS dataset. Tools with favourable performance characteristics can then be used to discover additional binding sites in closely related genomes or used in conjunction with experimental validation to improve the quality and comprehensiveness of existing TFBS databases. In RegulonDB, for example, of the methods tested Weeder, MEME and AlignACE present advantages over the other tools. AlignACE presents a more diverse list with more false positives whereas Weeder and MEME present true motif instances more often than the other tools. Motif prediction tools are composed of both a motif scoring function and a discrimination algorithm. The scoring function accepts a motif representation (e.g. a probability weight matrix) and then calculates the motif prediction candidates based on a discrimination function (e.g. maximum likelihood). Discrimination algorithms present a computational strategy to approximate the multiple sequence alignment of predicted binding positions relative to all multiple sequence alignments found in the background signal. Both Weeder and AlignACE have original scoring functions that could explain their utility on RegulonDB. MEME on the other hand uses expectation maximization as its discrimination algorithm. It could be that MEME benefits from this strategy over programs utilizing gibbs sampling. On the other hand, it is also likely that the predictions provided by these programs happen to be better over RegulonDB by random chance.

## Conclusion

In this paper we have presented a general method, MTAP, for evaluating cis-regulatory motif discovery tools. MTAP is novel and completely different from other approaches in that it allows both algorithm practitioners and users the flexibility to dynamically change attributes of data collection, algorithm parameters, and assessment. Our results indicate a clear need toward improvements in each of these areas. In our results, we explored four of the most commonly attributed factors to prediction accuracy: upstream file size, length of upstream sequences, TFBS density, and TFBS information content. The results obtained by MTAP in this assessment do not point toward any of these individual factors as playing a critical roll in finding TFBS. The results do indicate that the ratio of information content over upstream file size may have an influence on performance for some tools.

The primary innovation in MTAP is not that we produce additional tools or additional benchmarks, but it is that we produce a platform that can be used to improve tools and the benchmarking process. The results presented in this paper indicate that the methods used to prepare upstream data, the algorithm, the parameters, and the method used in evaluation all play important rolls in how we look at the cis-regulatory motif discovery problem.

In the past, many authors have dismissed bacterial regulatory motif detection as a far simpler problem than eukaryote regulatory motif detection (e.g. Xing *et. al *[35]). While it is true that the annotated bacteria regulatory modules do not have the same level of complexity and combinatorial control, our results indicate that even for this 'simple' problem, regulatory motif detection methods have substantial room for improvement.

Unlike other approaches, MTAP allows for the integration of regulatory regions from other species through an automated procedure. It remains to be seen which integration procedure and what combination of closely related and distantly related species improves performance for tools that incorporate regulatory regions from phylogentically related species. This is the subject of future work. At the moment, it does not appear that two closely related strains are enough to improve performance over conventional single sequence approaches. It would be interesting to extend our current implementation of MTAP and assess tools that integrate data from expression arrays and ChIP-chip arrays. Such approaches should lead to an increase in performance, but the parameters and procedures for this are not currently clear.

It is not currently understood what features of the data make the problem of finding TFBS so difficult. The key advantage of MTAP is that it allows us to explore these features and propose new models that are more accurate and robust. It is important to understand the performance characteristics of the models that have been proposed in the past before we integrate additional information. In this way, we can understand more completely if the relationships found by more sophisticated techniques are real or if they could have occurred by random chance. Further exploration into motif representation, motif scoring, and the relationship between binding sites is still necessary if we are to accurately predict regulatory binding sites on the computer.

## Competing interests

The authors declare that they have no competing interests.

## Authors' contributions

DQ, HA, and DB proposed the design of the system. KD implemented the prediction pipelines and parsers for each tool. DQ and KD integrated the databases. DQ and MS implemented the system and migrated the system to a clustered environment. DQ, KD and MS tested the system. DQ conducted the computational experiments and wrote the manuscript. All authors approved the final manuscript.

## References

[B1] Galas DJ, Eggert M, Waterman MS (1985). Rigorous pattern-recognition methods for DNA sequences. Analysis of promoter sequences from Escherichia coli. J Mol Biol.

[B2] Lawrence CE, Altschul SF, Boguski MS, Liu JS, Neuwald AF, Wootton JC (1993). Detecting subtle sequence signals: a Gibbs sampling strategy for multiple alignment. Science.

[B3] Thompson W, Rouchka E, Lawrence C (2003). Gibbs Recursive Sampler: finding transcription factor binding sites. Nucl Acids Res.

[B4] Bailey TL, Elkan C (1994). Fitting a mixture model by expectation maximization to discover motifs in biopolyme rs. Proc Int Conf Intell Syst Mol Biol.

[B5] Hertz G, Stormo G (1999). Identifying DNA and protein patterns with statistically significant alignments of multiple sequences. Bioinformatics.

[B6] Hertz GZ, Hartzell GW, Stormo GD (1990). Identification of consensus patterns in unaligned DNA sequences known to be functionally related. Comput Appl Biosci.

[B7] Liu X, Brutlag DL, Liu JS (2001). BioProspector: discovering conserved DNA motifs in upstream regulatory regions of co-expressed genes. Pac Symp Biocomput.

[B8] Hughes JD, Estep PW, Tavazoie S, Church GM (2000). Computational identification of cis-regulatory elements associated with groups of functionally related genes in Saccharomyces cerevisiae. J Mol Biol.

[B9] Workman CT, Stormo GD (2000). ANN-Spec: a method for discovering transcription factor binding sites with improved specificity. Pac Symp Biocomput.

[B10] Frith MC, Hansen U, Spouge JL, Weng Z (2004). Finding functional sequence elements by multiple local alignme nt. Nucleic Acids Res.

[B11] Pavesi G, Mauri G, Pesole G (2001). An algorithm for finding signals of unknown length in DNA sequences. Bioinformatics.

[B12] Pavesi G, Mereghetti P, Mauri G, Pesole G (2004). Weeder Web: discovery of transcription factor binding sites in a set of sequences from co-regulated genes. Nucleic Acids Res.

[B13] Che D, Jensen S, Cai L, Liu J (2005). BEST: Binding-site Estimation Suite of Tools. Bioinformatics.

[B14] Hu J, Yang Y, Kihara D (2006). EMD: an ensemble algorithm for discovering regulatory motifs in DNA sequences. BMC Bioinformatics.

[B15] Sinha S, Blanchette M, Tompa M (2004). PhyME: a probabilistic algorithm for finding motifs in sets of orthologous sequences. BMC Bioinformatics.

[B16] Siddharthan R, Siggia ED, van Nimwegen E (2005). PhyloGibbs: A Gibbs Sampling Motif Finder That Incorporates Phylogeny. PLoS Comput Biol.

[B17] Pavesi G, Zambelli F, Pesole G (2007). WeederH: an algorithm for finding conserved regulatory motifs and regions in homologous sequences. BMC Bioinformatics.

[B18] Roven C, Bussemaker HJ (2003). REDUCE: An online tool for inferring cis-regulatory elements and transcriptional module activities from microarray data. Nucleic Acids Res.

[B19] De Bie T, Monsieurs P, Engelen K, De Moor B, Cristianini N, Marchal K (2005). Discovering transcriptional modules from motif, chip-chip and microarray data. Pacific Symposium on Biocomputing Pacific Symposium on Biocomputing.

[B20] Tompa M, Li N, Bailey T, Church G, De Moor B, Eskin E, Favorov A, Frith M, Fu Y, Kent J (2005). Assessing computational tools for the discovery of transcription factor binding sites. Nature Biotechnology.

[B21] Sandve G, Abul O, Walseng V, Drablos F (2007). Improved benchmarks for computational motif discovery. BMC Bioinformatics.

[B22] Klepper K, Sandve G, Abul O, Johansen J, Drablos F (2008). Assessment of composite motif discovery methods. BMC Bioinformatics.

[B23] Hu J, Li B, Kihara D (2005). Limitations and potentials of current motif discovery algorithms. Nucleic Acids Research.

[B24] Salgado H, Gama-Castro S, Martinez-Antonio A, Diaz-Peredo E, Sanchez-Solano F, Peralta-Gil M, Garcia-Alonso D, Jimenez-Jacinto V, Santos-Zavaleta A, Bonavides-Martinez C (2004). RegulonDB (version 4.0): transcriptional regulation, ope ron organization and growth conditions in Escherichia coli K-12. Nucleic Acids Res.

[B25] Price M, Dehal P, Arkin A (2007). Orthologous Transcription Factors in Bacteria Have Different Functions and Regulate Different Genes. PLoS Computational Biology.

[B26] Quest D, Dempsey K, Shafiullah M, Bastola D, Ali H (2008). A Parallel Architecture for Regulatory Motif Algorithm Assessment. Hicomb: 2008.

[B27] Ishii T, Yoshida K-I, Terai G, Fujita Y, Nakai K (2001). DBTBS: a database of Bacillus subtilis promoters and transcription factors. Nucl Acids Res.

[B28] Munch R, Hiller K, Barg H, Heldt D, Linz S, Wingender E, Jahn D (2003). PRODORIC: prokaryotic database of gene regulation. Nucleic Acids Res.

[B29] Kazakov AE, Cipriano MJ, Novichkov PS, Minovitsky S, Vinogradov DV, Arkin A, Mironov AA, Gelfand MS, Dubchak I (2006). RegTransBase – a database of regulatory sequences and inte ractions in a wide range of prokaryotic genomes. Nucleic Acids Res.

[B30] Wingender E, Dietze P, Karas H, Knuppel R (1996). TRANSFAC: a database on transcription factors and their DNA binding sites. Nucleic Acids Res.

[B31] Wall DP, Fraser HB, Hirsh AE (2003). Detecting putative orthologs. Bioinformatics.

[B32] (2000). ELPH Manual from the Center for Bioinformatics and Computational Biology.

[B33] Bailey TL, Williams N, Misleh C, Li WW (2006). MEME: discovering and analyzing DNA and protein sequence motifs. Nucleic Acids Res.

[B34] Fawcett T (2006). An introduction to ROC analysis. ROC Analysis in Pattern Recognition.

[B35] Xing EP, Wu W, Jordan MI, Karp RM (2004). Logos: a modular bayesian model for de novo motif detection. J Bioinform Comput Biol.

